# Lipid-rich diet enhances L-cell density in obese subjects and in mice through improved L-cell differentiation

**DOI:** 10.1017/jns.2015.11

**Published:** 2015-05-20

**Authors:** Thomas Aranias, Alexandra Grosfeld, Christine Poitou, Amal Ait Omar, Maude Le Gall, Sylvie Miquel, Kévin Garbin, Agnès Ribeiro, Jean-Luc Bouillot, André Bado, Edith Brot-Laroche, Karine Clément, Armelle Leturque, Sandra Guilmeau, Patricia Serradas

**Affiliations:** 1 Inserm UMR_S 1138, Centre de Recherche des Cordeliers; Sorbonne universités, UPMC Univ Paris 06; Sorbonne Cités, UPD Univ Paris 05; F-75006, Paris, France; 2 Institut de Cardiométabolisme et Nutrition, ICAN, Pitié-Salpêtrière Hospital, Paris, France; 3 Sorbonne universités, UPMC Univ Paris 06, UMR_S ICAN U1166; Inserm; Nutriomic Team, F-75013, Paris, France; 4 Assistance Publique-Hôpitaux de Paris, Pitié-Salpêtrière Hospital, Heart and Metabolism Department, Nutrition Unit, 47–83 Boulevard de l'Hôpital, 75651 Paris Cedex 13, France; 5 Inserm UMR_S 1149, DHU Unity, UFR de Médecine Paris Diderot; Sorbonne Cités, UPD Univ Paris 05, F-75890, Paris, France; 6 INRA UMR 1319 Micalis, Interactions des Commensales et Probiotiques avec l'Hôte, F-78350 Jouy-en-Josas, France; 7 AgroParisTech, UMR 1319 Micalis, F-78350 Jouy-en-Josas, France; 8 Assistance Publique-Hôpitaux de Paris, Surgery Department, Ambroise Paré Hospital, Boulogne-Billancourt, France; 9 Institut Cochin, Inserm UMR_S 1016, CNRS UMR 8104, Sorbonne Cités, UPD Univ Paris 05; Endocrinology, Metabolism and Diabetes Department, F-75014; Paris, France

**Keywords:** High-fat diet, Intestine, Enteroendocrine cells, Gut hormones, BrdU, bromodeoxyuridine, CD, control diet, foxa1, forkhead box protein A1, foxa2, forkhead box protein A2, GIP, glucose-dependent insulinotropic polypeptide, GLP-1, glucagon-like peptide-1, HFD, high-fat diet, isl1, insulin gene enhancer protein-1, ngn3, neurogenin3, pax6, paired box protein-6, PYY, peptide YY

## Abstract

The enterohormone glucagon-like peptide-1 (GLP-1) is required to amplify glucose-induced insulin secretion that facilitates peripheral glucose utilisation. Alteration in GLP-1 secretion during obesity has been reported but is still controversial. Due to the high adaptability of intestinal cells to environmental changes, we hypothesised that the density of GLP-1-producing cells could be modified by nutritional factors to prevent the deterioration of metabolic condition in obesity. We quantified L-cell density in jejunum samples collected during Roux-en-Y gastric bypass in forty-nine severely obese subjects analysed according to their fat consumption. In mice, we deciphered the mechanisms by which a high-fat diet (HFD) makes an impact on enteroendocrine cell density and function. L-cell density in the jejunum was higher in obese subjects consuming >30 % fat compared with low fat eaters. Mice fed a HFD for 8 weeks displayed an increase in GLP-1-positive cells in the jejunum and colon accordingly to GLP-1 secretion. The regulation by the HFD appears specific to GLP-1-producing cells, as the number of PYY (peptide YY)-positive cells remained unchanged. Moreover, genetically obese *ob/ob* mice did not show alteration of GLP-1-positive cell density in the jejunum or colon, suggesting that obesity *per se* is not sufficient to trigger the mechanism. The higher L-cell density in HFD-fed mice involved a rise in L-cell terminal differentiation as witnessed by the increased expression of transcription factors downstream of neurogenin3 (*Ngn3*). We suggest that the observed increase in GLP-1-positive cell density triggered by high fat consumption in humans and mice might favour insulin secretion and therefore constitute an adaptive response of the intestine to balance diet-induced insulin resistance.

Despite their relative low density along the gastrointestinal epithelium (about 1 % of total cells), enteroendocrine cells constitute the largest population of endocrine cells in the organism^(^^1^^)^. Over recent years, the gut has emerged as a key player in metabolic control through the secretion of a large panel of enteropeptides in response to a meal. Indeed, the crucial role of intestinal hormones has been established in the control of food intake, energy expenditure, glucose homeostasis, lipid metabolism and a wide range of other metabolic functions^(^^1^^,^^2^^)^. This is illustrated by the phenotype of mice with total loss of enteroendocrine cells, which show growth retardation and high mortality at weaning^(^^3^^)^.

In order to ensure an adequate and physiological response following meal ingestion, hormonal secretion from the gut is finely tuned by nutrients^(^^4^^)^. Among enterohormones, glucagon-like peptide-1 (GLP-1) and glucose-dependent insulinotropic polypeptide (GIP), secreted by L- and K-cells, respectively, display an incretin effect through stimulation of insulin secretion in response to oral glucose^(^^2^^)^. In metabolic diseases, diminished GLP-1 levels in response to nutrients have been reported in some studies but are still controversial^(^^5^^,^^6^^)^. Moreover, the relative contribution of changes in differentiation, number and/or function of L-cells to the alteration of gut hormone secretion in response to a chronic unbalanced diet is still poorly understood. However, it is well established that L-cells are equipped to detect nutrients such as carbohydrates and lipids^(^^7^^)^ and their specific distribution along the gut from the absorptive small intestine to the microbiota-colonised large intestine highlights multiple possible controls by nutrients. In this context, the number of L-cells has been shown to increase in mice fed a prebiotic fibre-rich diet^(^^8^^)^, which might thereby improve the metabolic status of obese and type 2 diabetic subjects^(^^9^^)^. Although the molecular mechanism by which oligofructose modifies enteroendocrine L-cell density in the proximal colon remains to be characterised, the authors suggested an up-regulation of the differentiation process.

Intestinal epithelial cells are constantly renewed throughout the life span while preserving tissue homeostasis^(^^10^^)^. This requires a balance between cell proliferation, apoptosis and differentiation processes. A well-defined cascade of transcription factors controls the terminal differentiation of enteroendocrine cell subtypes. Following Notch signalling repression^(^^10^^)^ and consecutive Math 1 expression, which engages intestinal progenitors towards the secretory lineage^(^^11^^)^, transient expression of neurogenin3 (Ngn3) is then required to restrict secretory progenitors to the endocrine lineage^(^^12^^)^. This later will give rise to different cell subtypes such as D- (somatostatin), L- (GLP-1, PYY (peptide YY)), I- (cholecystokinin) and S- (secretin) cells. Foxa1/Foxa2 (forkhead box protein A1/A2) and NeuroD act downstream of Ngn3 to allow the commitment into the D- and L-cell or into the I- and S-cell phenotypes, respectively^(^^13^^)^. Late-acting transcription factors such as Pax4 (paired box protein-4), Pax6 (paired box protein-6) and Isl1 (insulin gene enhancer protein-1) regulate enterohormone gene transcription^(^^11^^)^ leading to different expression levels of GLP-1 and PYY in L-cells according to their location along the gut^(^^14^^,^^15^^)^. However, it is not known whether and how the nutritional environment alters transcription factors involved in enteroendocrine lineage in metabolic diseases.

In the present study, our hypothesis was that a diet enriched in lipid over carbohydrate contents could modulate the density and function of intestinal GLP-1-producing cells. We studied L-cell density in the jejunum of obese subjects according to their level of fat consumption. In mice, we deciphered the mechanisms by which high fat feeding could modulate L-cell density, GLP-1 secretion and the expression of transcription factors involved in enteroendocrine cell differentiation in the jejunum and colon.

## Experimental methods

### Human subjects and tissue samples

Morbidly obese individuals involved in a bariatric surgery programme were recruited by the Nutrition Department of Pitié-Salpêtrière Hospital (Reference Centre for the Medical and Surgery Care of Obesity, Paris, France). Obese subjects were aged between 24 and 64 years and met the criteria for bariatric surgery: BMI ≥ 40 kg/m^2^ (98 %) or BMI ≥ 35 kg/m^2^ combined at least with one co-morbidity (type 2 diabetes (55 %), hypertension (59 %) or dyslipidaemia (61 %)). Subjects were weight stable (i.e. variation of less than 3 kg) for at least 3 months before surgery and treated with antibiotics to prevent any *Helicobacter pylori infection*. Subjects did not demonstrate evidence of inflammatory diseases, infectious diseases, cancer, alcohol consumption, and did not display surgical complications during the 3 months after surgery. Of the twenty-seven diabetic subjects, twenty-two were taking antidiabetic medication: insulin (*n* 4); metformin (*n* 3); thiazolidinedione (*n* 2); sulfonylureas (*n* 1); metformin and sulfonylureas (*n* 6); insulin and metformin (*n* 2); metformin, sulfonylureas and thiazolidinedione (*n* 2); insulin, metformin and sulfonylureas (*n* 2); GLP-1 analogues or dipeptidyl peptidase-4 (DPP-IV) inhibitors (*n* 0). Both energy (kcal/d) and macronutrient intake (%/d) were recorded by a registered dietitian from patients’ 3 d food diaries 1–3 months before surgery. Obese subjects were asked to maintain usual food habits before surgery. The subjects could be classified into groups based on macronutrient consumption. A group of high fat and low carbohydrate eaters was constituted by subjects ingesting >30 % of energy as lipids and <50 % of energy as carbohydrates.

Subjects were fasted as required for the surgery (Roux-en-Y gastric bypass). Proximal jejunum samples (2 cm) were surgical wastes taken at 50 cm distal to the angle of Treitz by the same surgeon (J.-L. B.).

The study was conducted in accordance with the Helsinki Declaration and was recorded in a public trial registry (identity no. NCT00476658). Obese subjects provided a written informed consent, once the purpose of the study had been explained.

### Animals and diets

C57BL6/J male mice, aged 6 weeks, as well as 8-week old male *ob/ob* and age-matched control mice were obtained from Janvier and acclimatised in a conventional animal facility. C57BL6/J mice were fed *ad libitum* for up to 8 weeks either a control diet (CD) containing 4·2 % fat (w/w) (Sniff diet E15000–047), or a high-fat diet (HFD) containing 34 % fat (w/w) (Sniff diet E15741–347), the fat ratio being increased at the expense of carbohydrate, as usual (Table 1). *Ob/ob* and control mice were fed *ad libitum* with a standard diet (Table 1). Body weight was measured weekly as well as glycaemia in fed mice using an Accu-Chek^®^ glucometer.

**Table 1.** Composition of mouse diets*

Diet…	CD			HFD			Standard diet		
	w/w %	Energy (kcal/100 g diet)	Energy (kJ/100 g diet)	w/w %	Energy (kcal/100 g diet)	Energy (kJ/100 g diet)	w/w %	Energy (kcal/100 g diet)	Energy (kJ/100 g diet)
Lipids	4·2	37·8	158·2	34·0	306·0	1281·1	5·1	45·9	192·0
Starch	46·8	187·2	783·2	2·2	8·8	36·8	34·0	136·0	569·0
Oligosaccharides	10·8	43·2	180·7	22·4	89·6	375·1	17·7	70·8	296·2
Proteins	20·8	83·2	348·1	24·1	96·4	403·3	21·4	85·6	358·2
Fibres	5·0			6·0			4·0		
Ash	5·6			6·1			5·7		
Water	6·8			5·2			12·1		
Total		351·4	1470·2		500·8	2095·3		338·3	1415·4

CD, control diet; HFD, high-fat diet.

* The CD and HFD were used for the diet-induced obesity study whereas the standard diet was administered to the *ob/ob* mice and age-matched lean mice. Nutrient diet compositions are expressed as percentage weight and energy content.

Experimental procedures conforming to the French guidelines for animal studies were approved by the Regional Animal Care and Use Committee (CREEA Ile-de-France no. 3, agreement no. p3/2008/042).

### Oral glucose tolerance test and plasma measurements

After 2 and 8 weeks of diet, mice were fasted overnight (16 h). For the oral glucose tolerance test (OGTT), glycaemia was measured immediately before or 15, 30, 60, 90 and 120 min after an oral glucose load (4 g/kg).

Other plasma parameters were quantified in blood samples collected from the tail vein in EDTA-coated tubes in the presence of dipeptidyl peptidase-4 (DPP-4) inhibitor (Millipore), 15 min after a glucose bolus (4 g/kg). Plasma insulin, glucagon and total GIP were measured in 20 μl using a multiplex immunoassay kit (Millipore) and performed with Luminex. Plasma total GLP-1 was determined in 50 μl using an ELISA kit (Millipore).

Total and HDL-cholesterol, TAG, NEFA and hydroxybutyrate were measured in plasma samples using a multi-parametric automated Olympus AU400 chemistry analyser.

### mRNA extraction and quantitative RT-PCR analysis

Mouse jejunal and colonic mucosa were scraped, snap-frozen and kept at –80 °C until RNA extraction using TRIzol^®^ reagent (Invitrogen). Between 2 and 4 μg RNA were reverse transcribed with 200 units of RT using the Superscript II kit (Invitrogen) according to the manufacturer's recommendations. Quantitative RT-PCR analyses were performed with a LightCycler 480 instrument (Roche Applied Science) and cDNA was amplified using SYBR^®^ Green PCR Master Mix (Roche Applied Science) and specific primers (online Supplementary Table S1). The relative abundance of each amplified product was expressed in arbitrary units as a ratio of the target transcript normalised to murine TATA-binding protein (TBP) mRNA level.

### Cell isolation and flow cytometry

Mouse proximal jejunum (10 cm) was collected, washed in PBS, and cut in 2–3 mm pieces in chelating buffer (PBS, 1 mm-DTT (dithiothreitol), 5 mm-EDTA). Jejunal fragments were thoroughly vortexed before and after two incubations (20 min at 37 °C under agitation) in the chelating buffer. Epithelial cell suspension was then filtered on a 70 μm cell strainer on top of complete medium (Roswell Park Memorial Institute (RPMI)-1640 medium, fetal calf serum (FCS) 10 %, penicillin-streptomycin (PS) 1 %). After centrifugation, cells were re-suspended in complete medium. After fixing in 4 % paraformaldehyde (PFA), 10^6^ cells were incubated 30 min with the primary GLP-1 antibody (no. sc-7782; Santa Cruz) or control IgG (no. sc-2028; Santa Cruz) and a Cy5-coupled secondary antibody (Jackson Immunoresearch) diluted in a permeabilisation buffer (PBS, FCS 2 %, saponin 0·1 %). Stained cells in suspension were analysed with a BD LSRII cytometer and expressed as a percentage of total epithelial cells.

### Immunohistochemistry

Human jejunum samples were fixed in alcohol–formalin–acetic acid and embedded in paraffin wax. Immunofluorescence staining was performed on 6 μm tissue sections and analysed by confocal microscopy (LSM710; Zeiss; 40× oil lens, 0·8 μm depth of field). GLP-1(7–36) amide antibody (no. T4057; 1/1,000 Peninsula Bachem) was used to locate human L-cells. Primary antibodies were revealed with secondary antibodies coupled to cyanin 2 (1/400 Jackson). GLP-1-positive L-cells per subject were expressed per 100 villi.

Mouse proximal jejunum and colon were collected, fixed in a 4 % PFA solution and embedded in paraffin; 5 μm sections were rehydrated. Antigen retrieval was performed in a pH 6 citrate buffer (10 min at 97 °C), endogenous peroxidase activity was blocked in 3 % H_2_O_2_ (5 min) and unspecific binding sites were blocked with 5 % goat serum. L-enteroendocrine cells were labelled using either an anti-GLP-1 (no. T-4057; Peninsula) or an anti-PYY antibody (no. ab22663; Abcam). A cleaved caspase-3 antibody (no. 9579; Cell Signaling) labelled apoptotic cells. As GLP-1 positive cells are rare and GLP-1 and caspase-3 double-positive cells even more rare events, total apoptotic epithelial cells were evaluated. Signals were revealed using the biotinylated secondary antibody/streptavidin-HRP (horseradish peroxidase) amplification system (BioSpa) and DAB (diaminobenzidine; Dako). After dehydration, sections were bathed in Histoclear and mounted in Eukitt (O. Kindler). Images were obtained using a light microscope (Leica Leitz DMRB) and acquired with QWin software (Leica Microsystems).

### Cell proliferation

For cell proliferation studies, mice were fed *ad libitum* either the CD or HFD for 2 weeks. Bromodeoxyuridine (BrdU) was added to drinking water (80 mg/100 ml) for 6 d. The proximal jejunum and colon were collected and processed as described above. Proliferating L-cells were identified by a double staining of GLP-1 and BrdU (no. ab6326; Abcam). Signals were revealed using Alexa Fluor 488 and Alexa Fluor 546 coupled secondary antibodies, respectively (Molecular Probes) and nuclei were stained with DAPI (4′,6-diamidino-2-phenylindole). Sections were mounted in Fluoprep (bioMérieux). Images were obtained by confocal microscopy (LSM710; Zeiss) and acquired with Zen software (Zeiss).

### Data presentation and statistical analysis

Statistical analyses were performed using a non-parametric Mann–Whitney test or χ^2^ analysis for qualitative parameters (Prism5; GraphPad) with a threshold for significance of *P* < 0·05. All data are expressed as mean values with their standard errors.

## Results

### Association of high-fat consumption with increased glucagon-like peptide-1-positive cells in the jejunum of obese subjects

L-cell density was investigated in forty-nine obese subjects according to their macronutrient consumption. The mean daily food intake in this group was estimated at 2100 (sem 100) kcal/d (8786 (sem 418) kJ/d); thirty-three of the forty-nine (67·3 %) obese subjects consumed >30 % lipids (FAO recommendations 15–30 % of total energy intake) and <50 % carbohydrates (FAO recommendations 55–75 %) and we therefore classified them as high fat eaters. The others were classified as low fat eaters (Table 2).

**Table 2.** Bioclinical parameters and endocrine L-cell density in obese subjects (Mean values with their standard errors, or numbers of subjects and percentages)

	Low fat eaters (*n* 16)		High fat eaters (*n* 33)		
	Mean	sem	Mean	sem	P*
Sex (*n*)					0·09
Female	15		24		
Male	1		9		
Age (years)	48·5	2·4	42·9	2·0	0·10
BMI (kg/m^2^)	49·0	2·0	50·4	1·1	0·28
Diabetes					0·18
Yes					
*n*	11		16		
%	69		48		
No					
*n*	5		17		
%	31		52		
Energy intake†					<0·01
kcal/d	1805	157	2212	111	
kJ/d	7557	657	9261	464	
Proteins (%)	18·2	1·1	20·0	0·6	0·17
Lipids (%)	27·2	1·0	39·5	0·8	<0·0001
Carbohydrates (%)	54·4	1·7	40·5	1·0	<0·0001
L-cell density (cells/100 villi)	35	18	57	4	<0·005

* By Mann–Whitney or χ^2^ analysis.

† Energy intake was recorded using a food questionnaire and allowed classification into high fat and low fat eaters.

GLP-1-positive cell density in the jejunum varied from 35 (sem 18) cells/100 villi in low fat eaters compared with 57 (sem 4) cells/100 villi in high fat eaters (*P* = 0·0029) (Table 2), suggesting that high fat consumption increased L-cell density in the jejunum of obese subjects.

### Chronic high-fat diet enhances glucagon-like peptide-1 but not glucose-dependent insulinotropic polypeptide secretion in mice

To decipher the mechanism underlying enhanced L-cell density in high fat eater subjects, we fed mice with a HFD with a clear imbalanced proportion between lipid and carbohydrate (60 % lipids, 20 % carbohydrates). We first analysed their metabolic parameters during chronic feeding of the HFD. As expected, the HFD significantly enhanced body weight, total and HDL plasma cholesterol as early as 2 weeks after commencement of the diet. Body-weight gain in HFD mice was further exacerbated up to 8 weeks (Table 3). After 2 and 8 weeks of the diet, fasted blood glucose was higher in HFD mice than in CD mice (*P* < 0·001 and *P* < 0·01, respectively (Table 3), and the oral glucose tolerance test (OGTT) revealed as expected a gradual glucose intolerance (data not shown). Therefore, we investigated glucose-regulated insulin and glucagon secretions during HFD feeding. Glucose-induced insulin secretion rose in both CD and HFD mice, and the increase above basal level in the HFD mice was twice that observed in the CD mice after 2 and 8 weeks of diet. In parallel, glucagon secretion was inhibited by a glucose bolus in the CD mice, but not in the HFD mice after 2 and 8 weeks of diet (*P* < 0·01) (Table 3). Despite higher insulin secretion, blood glucose remained high in mice after the HFD (*P* < 0·01) as expected, suggesting an insulin-resistant state (Table 3).

**Table 3.** Metabolic parameters of mice fed the control diet (CD) or the high-fat diet (HFD)† (Mean values with their standard errors)

	2 weeks				8 weeks			
	CD		HFD		CD		HFD	
	Mean	sem	Mean	sem	Mean	sem	Mean	sem
Body weight (g)	24·8	0·5	27·5**	0·3	26·2	0·7	32·0***	0·4
Fed blood glucose (mmol/l)	8·49	0·67	10·60	0·39	9·82	0·44	12·21**	0·44
Fasted blood glucose (mmol/l)	4·77	0·17	7·49***	0·61	3·94	0·28	8·77**	0·50
Change in insulin (pg/ml)	466	78	938	202	528	68	919*	170
Change in glucagon (pg/ml)	−24	5	6**	5	−23	3	5**	7
Total cholesterol (mmol/l)	2·55	0·12	3·42**	0·28				
HDL (mmol/l)	1·71	0·08	2·29*	0·19				
TAG (mmol/l)	0·73	0·05	0·61	0·07				
NEFA (mmol/l)	0·55	0·06	0·49	0·06				
Hydroxybutyrate (mmol/l)	0·11	0·01	0·12	0·02				

Mean value was significantly different from that of the CD group: * *P* < 0·05, ** *P* < 0·01, *** *P* < 0·001 (non-parametric Mann–Whitney test).

† Body weight and fed blood glucose concentrations were measured weekly at 09.00 hours (*n* 8–16). Fasted blood glucose was measured after 16 h fasting. Changes in insulin and glucagon were calculated by the difference between glucose-stimulated minus basal plasma levels (*n* 8). Total and LDL-cholesterol, TAG, NEFA and hydroxybutyrate were measured in plasma of fasted mice after the oral glucose challenge (*n* 4).

**Fig. 1.**
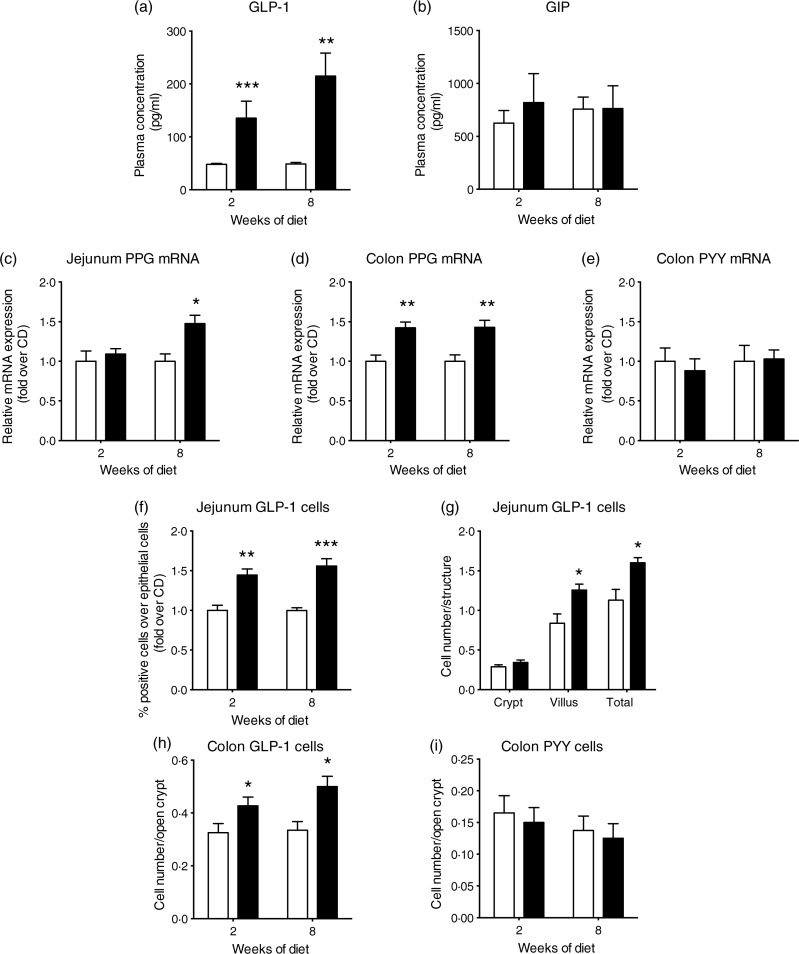
High-fat diet (HFD; ■) consumption increases glucose-stimulated glucagon-like peptide-1 (GLP-1) secretion and GLP-1 cell density in the intestine of mice. Mice fed a control diet (CD; □) or the HFD for 2 or 8 weeks were sampled 15 min after an oral glucose test (4 g/kg) for the measurement of plasma total GLP-1 (a) and plasma glucose-dependent insulinotropic polypeptide (GIP) (b) concentrations (*n* 5–8). mRNA analysis (c–e) and enteroendocrine cell quantification (f–i) of mice fed the CD or HFD. mRNA expression (*n* 7–8) of preproglucagon (PPG) (c, d) and peptide YY (PYY) (e). Fluorescence activated cell sorting (FACS) quantification (f) of GLP-1-positive cells relative to total isolated epithelial cells (*n* 7–10). Immunostaining quantification (g) of GLP-1 cells in the jejunum of mice after 8 weeks of diet (*n* 4). Immunostaining quantification (h) of GLP-1 cells in the colon of mice (*n* 4). Immunostaining quantification (i) of PYY cells in the colon of mice (*n* 4). Values are means, with standard errors represented by vertical bars. Mean value was significantly different from that of the CD group: * *P* < 0·05, ** *P* < 0·01, *** *P* < 0·001 (non-parametric Mann–Whitney test).

In this context of altered glucose-mediated insulin secretion in HFD mice, we then investigated the secretion of incretins, GLP-1 and GIP. Basal total plasma GLP-1 levels were below detection (13·5 pg/ml) both in CD and HFD mice. Glucose-stimulated GLP-1 plasma levels in HFD mice were three and four times higher than in CD mice after 2 weeks (*P* < 0·001) and 8 weeks (*P* < 0·01), respectively (Fig. 1(a)). Remarkably, there was no statistical difference in GIP plasma levels between both groups (Fig. 1(b)). These results suggest that an increased GLP-1 secretion could contribute in part to the hyperinsulinaemia observed in HFD mice, together with other factors such as insulin resistance. As the intestinal epithelium displayed a high rate of renewal, we addressed whether these changes related to an enhanced secretion of GLP-1 per cell and/or to an expanded enteroendocrine L-cell population.

### High-fat diet increases the number of enteroendocrine L-cells along the intestine in mice

L-cells can synthesise preproglucagon (GLP-1 precursor) and PYY mRNA. Their levels were investigated in the jejunal and colonic mucosa of either CD- or HFD-fed mice. In the jejunum, preproglucagon mRNA level was 1·5-fold higher in the HFD than in CD mice after 8 weeks (*P* < 0·05) (Fig. 1(c)). As expected from earlier studies, PYY mRNA was not detected in the jejunum in the CD and HFD mice (results not shown). In the colon, a 1·5-fold increase in preproglucagon mRNA levels was also observed after 2 and 8 weeks in the HFD mice compared with the CD mice (*P* < 0·01) (Fig. 1(d)), whereas PYY mRNA levels remained unaffected (Fig. 1(e)). A significant 1·4-fold increase in GLP-1 positive cells over jejunal epithelial cells in the HFD compared with CD mice (*P* < 0·01, 2 weeks; *P* < 0·001, 8 weeks of diet) (Fig. 1(f)) was measured by flow cytometry (online Supplementary Fig. S1(a)). Immunohistochemistry analysis demonstrated that the increase in GLP-1-positive cells occurs in the villus compartment (Fig. 1(g) and online Supplementary Fig. S1(b)) without change in villus length (online Supplementary Fig. S2). Moreover, a 1·3- and 1·5-fold increase in GLP-1-positive cells was observed in the colon of the HFD mice (after 2 and 8 weeks, respectively) (*P* < 0·05) (Fig. 1(h) and online Supplementary Fig. S1(c)) but no difference in PYY-positive cell density in the colon of mice was detected (Fig. 1(i) and online Supplementary Fig. S1(d)). All together, these data suggest that the HFD induced a rise in plasma GLP-1 at least in part by the expansion of intestinal L-cell density. Moreover, a specific role of the HFD on GLP-1 expression by enteroendocrine L-cells is highlighted since PYY expression remained unaffected.

**Fig. 2.**
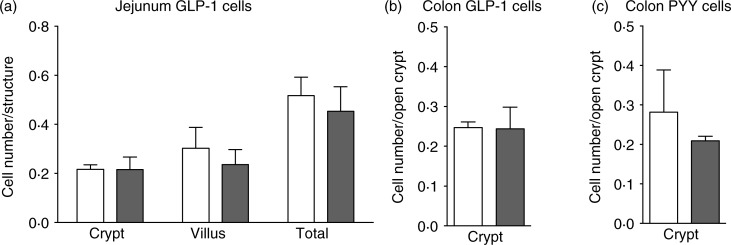
No modification of enteroendocrine cell density in genetically obese *ob/ob* (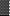) mice. Enteroendocrine cell density in lean (wild type; □) and *ob/ob* mice fed the control diet. Quantification after immunostaining (*n* 3–4) of glucagon-like peptide-1 (GLP-1) cells in the jejunum (a), GLP-1 cells in the colon (b) and peptide YY (PYY) cells in the colon (c). Values are means, with standard errors represented by vertical bars. Statistical analysis was performed using a non-parametric Mann–Whitney test; there were no significant differences between the groups.

We next determined whether this feature was caused by nutritional factors, hormonal alterations or modification of gut microbiota-derived SCFA, which accompany obesity. GLP-1-positive cell density was studied in genetically obese *ob/ob* leptin-deficient mice fed a chow diet. Using this model, the impact of increased body weight can be distinguished from the impact of high-fat diet consumption. In *ob/ob* mice, body weight, total and HDL-cholesterol plasma levels were similar to those of HFD mice (data not shown) but no change in GLP-1- or PYY-positive cell density was observed (Fig. 2(a)–(c)). Moreover, parallel evaluation of SCFA content and composition in HFD-fed mice showed no difference in total caecal SCFA, or in acetate, propionate and butyrate contents between HFD and CD mice, indicating that mice were able to digest similarly the polysaccharides present in the two diets (online Supplementary Fig. S3). Altogether these data suggest that neither metabolic nor gut microbiota alterations but rather diet was responsible for increased GLP-1-positive cell density following HFD consumption.

### High-fat diet feeding promotes enteroendocrine L-cell lineage differentiation in mice

**Fig. 3.**
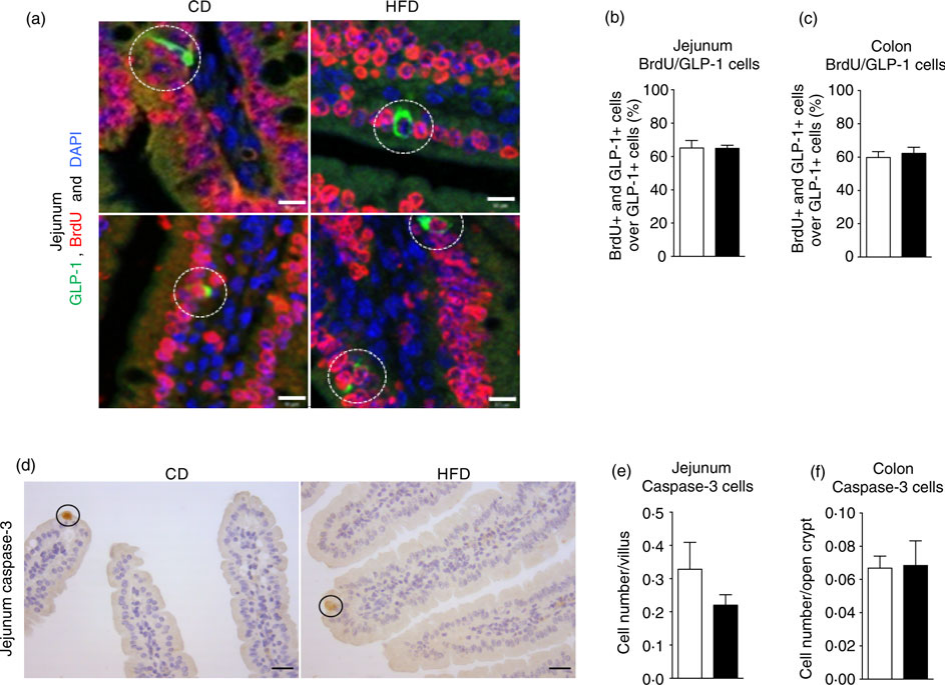
High-fat diet (HFD; ■) consumption does not modify enteroendocrine cell proliferation or apoptosis in mice. Representative double immunofluorescent staining (*n* 7–8) of glucagon-like peptide-1 (GLP-1) (green) and bromodeoxyuridine (BrdU) (red) with DAPI (4′,6-diamidino-2-phenylindole) staining (blue) in the jejunum (a) of mice fed a control diet (CD; □) or the HFD for 2 weeks. The lower panels of (a) show GLP-1 and BrdU double-positive cells whereas the upper panels of (a) show GLP-1-positive BrdU-negative cells (highlighted with dotted circles). The scale bar represents 10 μm. Quantification as percentage of proliferating GLP-1 cells over total GLP-1 cells in the jejunum (b) and colon (c). Representative immunostaining (*n* 6–8) of cleaved caspase-3 positive cells in the jejunum (d) of mice fed the CD or HFD for 2 weeks. The scale bar represents 20 μm. Quantification of stained cells in the jejunum (e) and colon (f). Values are means, with standard errors represented by vertical bars. Statistical analysis was performed using a non-parametric Mann–Whitney test; there were no significant differences between the groups.

As the enteroendocrine L-cell density relies on the balance between cell proliferation, differentiation and apoptosis, we next determined whether a higher density of L-cells in response to the HFD could result from modulation of one of these processes. The rate of L-cell proliferation was therefore evaluated as the ratio of GLP-1 and BrdU double-stained cells over total GLP-1-positive cells. Most epithelial cells were BrdU positive since their lifetime was shorter than the 6 d duration of BrdU treatment (Fig. 3(a)–(c) and online Supplementary Fig. S4(a)). By contrast, the longer enteroendocrine cell lifetime was illustrated by the presence of BrdU-negative GLP-1-positive cells in both the jejunum (Fig. 3(a)) and colon (online Supplementary Fig. S4(a)). The HFD had no effect on this ratio in the jejunum (Fig. 3(b)) or colon (Fig. 3(c)), indicating that proliferation of L-cell progenitors was not likely to explain the increase in L-cell density. In accordance with this result, no change was observed in jejunum crypt length (online Supplementary Fig. S2). Intestinal cell apoptosis evaluation performed by caspase-3-positive labelling (Fig. 3(d)–(f) and online Supplementary Fig. S4(b)) indicated a similar abundance of activated caspase-3-positive cells in the jejunum (Fig. 3 (d) and (e)) and colon (Fig. 3(f) and online Supplementary Fig. S4(b)), between CD and HFD mice, showing identical apoptosis in both groups. Therefore, we investigated whether HFD feeding could modulate the L-cell differentiation programme.

**Fig. 4.**
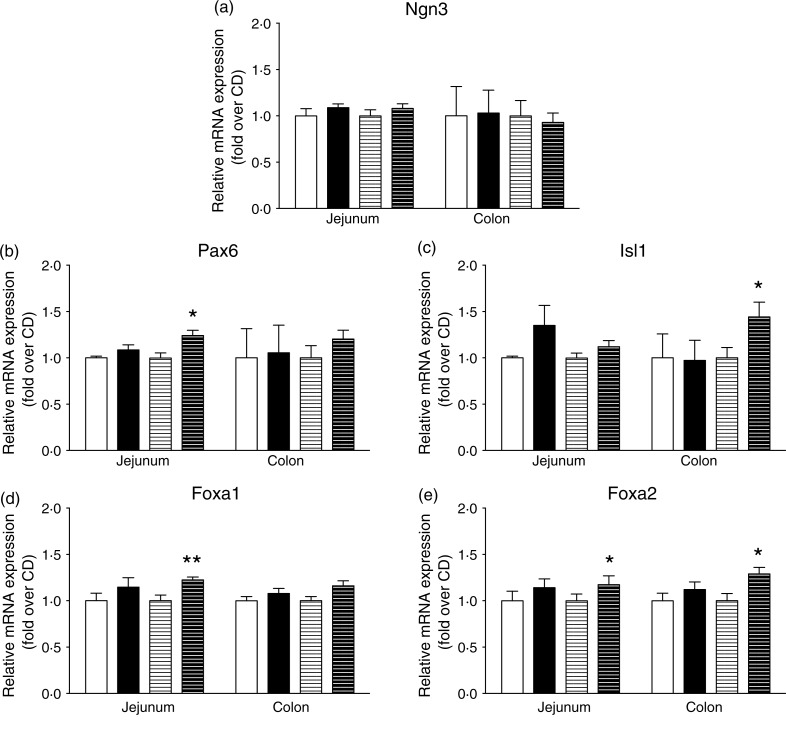
High-fat diet (HFD) consumption increases the mRNA abundance of transcription factors involved in L-enteroendocrine cell differentiation downstream of neurogenin3 (Ngn3). mRNA expression of mice fed a control diet (CD) for 2 weeks (□) or 8 weeks (=) or the HFD for 2 weeks (■) or 8 weeks (≡) were quantified by quantitative PCR and expression was normalised to TATA-binding protein (TBP). mRNA expression (*n* 6–8) of Ngn3 (a), paired box protein-6 (Pax6) (b), insulin gene enhancer protein-1 (Isl1) (c), forkhead box protein A1 (Foxa1) (d) and forkhead box protein A2 (Foxa2) (e). Values are means, with standard errors represented by vertical bars. Mean value was significantly different from that of the CD group: * *P* < 0·05, ** *P* < 0·01 (non-parametric Mann–Whitney test).

Engagement of intestinal secretory progenitors in the enteroendocrine lineage requires combined expression of transcription factors. As indicated in Fig. 4(a), intestinal *Ngn3* expression was not modified by the HFD. Interestingly, jejunal *Pax6* and colonic *Isl1* expression, two key transcription factors for preproglucagon expression, were enhanced by 20 % (*P* < 0·05) and 40 % (*P* < 0·05), respectively, in HFD as compared with CD mice after 8 weeks (Fig. 4(b)–(c)) (*P* < 0·05). Concomitantly, the HFD enhanced mRNA levels of *Foxa1* and *Foxa2* transcription factors, known regulators of *Pax6* and *Isl1* expression (Fig. 4 (d) and (e)). Indeed, *Foxa1* expression was increased by 20 % in the jejunum (Fig. 4(d)) (*P* < 0·01) and *Foxa2* expression by 30 % in the jejunum and colon (Fig. 4(e)) (*P* < 0·05). This higher expression of the transcription factors involved in GLP-1-producing cell differentiation downstream of *Ngn3* in response to the HFD was consistent with an enhanced L-cell population.

## Discussion

The present study shows for the first time that high fat/low carbohydrate consumption increases L-cell density in the jejunum of morbidly obese subjects. We confirmed these results in the jejunum, extended it to colonic samples from HFD-fed mice and further correlated this alteration with sustained GLP-1 secretion. Follow-up investigations regarding mechanisms involved in this process demonstrated that increased L-cell density triggered by HFD consumption implicates their terminal differentiation rather than alteration of their proliferation or apoptosis. Moreover, we suggest that neither obesity (in a model of leptin deficiency) nor associated changes in gut microbiota-derived SCFA are sufficient to increase L-cell density.

In a meta-analysis, plasma levels of the enterohormone GLP-1 have been linked to factors such as age and BMI rather than being a common feature of type 2 diabetes^(^^6^^)^. Because diabetes is often associated with obesity and unbalanced diet consumption, we investigated the effect of HFD feeding on plasma total GLP-1. In the present study, we showed a 3- to 4-fold increase in GLP-1 secretion in HFD mice in response to an oral glucose challenge. Our data slightly contrast with the decreased level of active GLP-1 previously reported in HFD-fed mice. However, this was associated with an elevated concentration of ileal and colonic GLP-1 content in those mice^(^^16^^)^. In agreement with the present study, a diet enriched in monounsaturated fats was reported as a powerful GLP-1 secretagogue, in lean rats^(^^17^^)^, suggesting an important role of diet composition in endogenous GLP-1 production.

We propose that enhanced GLP-1 secretion in HFD-fed mice is at least partly due to increased L-cell density in the gut. Indeed, modulation of nutrient sensing or absorptive capacities as well as differentiation programme by intestinal cell subtypes in response to changes in nutrient provision have been suggested to warrant gut adaptation to environmental needs^(^^18^^)^. Interestingly, only a few studies have reported that enteroendocrine cells might adjust to their nutritional environment during embryonic development or at the adult stage. Indeed, enteroendocrine cells display plasticity for hormone production at weaning in herbivores^(^^19^^)^. Moreover, the consumption of fibre-rich diet has been shown to increase L-cell number in mice^(^^8^^)^. In the present study, we demonstrated that L-cell density is increased in obese subjects eating a high-fat/low-carbohydrate diet, suggesting an *in vivo* modulation of these cells by macronutrients in human subjects. The HFD also triggered a rise in L-cell density in mice, as previously reported in rats^(^^20^^)^. A paper published concommitantly to our study also suggested an increase in L-cell density in HFD mice^(^^21^^)^. The effects of the HFD in mice might be due, as in human subjects, to the high lipid/low carbohydrate content but we cannot exclude the impact of the variable oligosaccharide proportion between the CD and HFD. A recent publication in rats reported opposite results^(^^22^^)^ and showed that a HFD reduced L-cell differentiation; however, biological tools used in this study remain to be validated to sustain such conclusions.

In the present work, we also elucidated the molecular mechanisms involved in enhanced L-cell number induced by high fat/low carbohydrate consumption. We showed that the initial stage of enteroendocrine cell differentiation is not affected as *Ngn3* mRNA levels remained unchanged in HFD mice^(^^12^^)^. In contrast, higher expression of *Foxa1, Foxa2, Pax6* and *Isl1* under HFD feeing suggests a potential regulation of L-cell terminal differentiation specifically^(^^11^^,^^13^^)^. In addition, up-regulation by the HFD of both *Pax6* and *Isl1*, which directly bind consensus sequences on preproglucagon promoter^(^^23^^,^^24^^)^, is consistent with the increased preproglucagon expression observed here. All together, the specific increase in GLP-1-producing cell differentiation in response to the HFD illustrates a potential intestinal tissue adaptation to the nutritional environment and could favour the control of energy homeostasis perturbed by the HFD.

In the high fat/low carbohydrate group, the amount of energy intake was significantly higher than in the control group, suggesting that the alteration in L-cell density may be due to other parameters than dietary fat amounts. Therefore, among the main characteristics associated with chronic HFD consumption^(^^25^^)^, we investigated whether increased body weight, and metabolic imbalance could underlie enhanced L-cell number. In *ob/ob* mice, which display increased body weight and metabolic alterations similar to those observed in HFD mice, GLP-1-positive cell density was not modified. These leptin-deficient mice have a higher energy intake compared with lean animals^(^^26^^)^; however, they did not show any modulation in L-cell number. This suggests that such common obesity features might not be sufficient *per se* to alter L-cell density. Nevertheless, it would be interesting to investigate the effect of leptin on L-cell density but our data on low- and high-fat eater obese subjects, known to be leptin resistant^(^^27^^)^, emphasise the role of diet composition on L-cell plasticity. Inasmuch as bile acid excretion is stimulated in response to HFD feeding^(^^28^^)^ and because several studies have shown that bile acids could stimulate GLP-1 secretion^(^^29^^,^^30^^)^, we cannot exclude an indirect effect of lipids through increased bile acids on L-cell secretion and/or density in our HFD mouse model.

SCFA, which are produced by gut microbiota from dietary indigestible complex carbohydrates, have been reported to modulate both GLP-1 secretion and L-cell differentiation^(^^8^^,^^31^^–^^33^^)^. Gut microbiota changes are now emerging as a functional consequence to HFD consumption, independently of obesity^(^^34^^)^. Here, we showed no alteration in SCFA caecal content in our HFD mouse model. The similar proportion of fibres provided in both diets (mostly low-fermentable cellulose) might explain this unchanged amount of SCFA production in HFD mice compared with CD mice. In addition, 2 weeks of HFD feeding did not modify gene expression of the SCFA receptors GPR41 and GPR43 (data not shown), whereas GLP-1 secretion and L-cell density were already increased. This suggests that SCFA are probably not responsible for the changes in L-cell density in the HFD mouse model.

As an alternative mechanism responsible for enhanced L-cell differentiation induced by HFD feeding, we suggest that lipids might favour changes in proliferation or metabolism of progenitor cells in response to the shift in provided energy substrates. Indeed, *n*-3 PUFA have been reported in rat colonocytes to suppress proliferation and increase differentiation and cell apoptosis, allowing crypt structure maintenance^(^^35^^)^. In this context, activation of the transcription factors, PPAR, could underlie the L-cell plasticity induced by lipids. The HFD used in the present study contained more than 50 % of unsaturated fat as a source of lipids, which are known as a preferential ligand for PPARβ/δ^(^^36^^,^^37^^)^. Moreover, total PPARβ/δ invalidation in mice resulted in decreased GLP-1 expression and secretion, as a result of the down-regulation of the preproglucagon promoter activity^(^^38^^)^. Therefore, HFD activation of PPARβ/δ might be involved in the increased GLP-1-positive cell density in the intestine, underlining a new role for this transcription factor in enteroendocrine cell lineage.

In conclusion, the present study shows that high fat/low carbohydrate feeding modulates intestinal cell plasticity and stimulates L-cell differentiation to enhance GLP-1 secretion. This progressive increment of circulating levels of GLP-1 over time might represent an adaptive stimulus for insulin secretion at early stages of a lipid-enriched diet. At later stages, this overstimulation of insulin secretion might ultimately result in an alteration in glucose metabolism and diabetes development.

## Supplementary material

For supplementary material accompanying this paper visit http://dx.doi.org/10.1017/jns.2015.11. click here to view supplementary material
