# Supplementary material for: Lipid-rich diet enhances L-cell density in obese subjects and in mice through improved L-cell differentiation
Source: J Nutr Sci. 2015 May 20;4:e22. doi: 10.1017/jns.2015.11 (PMC4459237; doi:10.1017/jns.2015.11)
Supplement: Supplementary file 1 [file S2048679015000117sup001.zip › S2048679015000117sup006.doc]

**SUPPLEMENTARY DATA**

**EXPERIMENTAL METHODS**

**Measurement of crypt and villus lengths in mice**

Jejunum villus and crypt lengths were measured using ImageJ software. Measurements were made on a mean of >40 longitudinally complete villi per mouse. Villus and crypt lengths were expressed as the distance from the crypt-villus junction to the villus top and the crypt-villus junction to the crypt bottom, respectively.

**Measurement of scfa caecum content in mice**

Volatile Short-Chain Fatty Acid (SCFA) concentrations were determined after water extraction of acidified caecal contents using gas liquid chromatography (Nelson 1020, Perkin-Elmer, St Quentin en Yvelines, France) .

**TABLE AND FIGURE LEGENDS**

**Sup. Table 1. Oligonucleotide sequences used for qPCR analysis.**

**Sup. Figure 1. L-cell abundance by FACS and immunostaining of mice fed control diet (CD) or high-fat diet (HFD).**

Cytograms of isolated epithelial jejunum GLP-1 stained cells (A) and representative immunostaining of GLP-1 in the jejunum (B) and the colon (C) and of PYY in the colon (D) of mice fed a control (CD) or high fat diet (HFD). Scale bar represents 20 μm.

**Sup. Figure 2. High-fat diet consumption does not modify the jejunal villus and crypt lengths in mice.**

Jejunal villus and crypt lengths of mice (n=7-8) fed control (CD) or high-fat diet (HFD) for 2 weeks. Values are mean ± SEM. Statistical analysis was performed using a non parametric Mann-Whitney test ns: non significant *vs* CD.

**Sup. Figure 3. High-fat diet consumption does not modify SCFA caecal content in mice.**

Caecal concentration (n=4) of total SCFAs (A), acetate (B), propionate (C) and butyrate (D) in mice fed control (CD) or high-fat diet (HFD) for 2 or 8 weeks. Values are mean ± SEM. Statistical analysis was performed using a non parametric Mann-Whitney test *p<0.05; ns: non significant *vs* CD.

**Sup. Figure 4. High-fat diet consumption does not modify enteroendocrine cell proliferation or apoptosis in the colon of mice.**

Representative double immunofluorescent staining (n=7-8) of GLP-1 (green) and BrdU (red) with DAPI staining (blue) in colon **(A)** of mice fed control (CD) or high-fat diet (HFD) for 2 weeks. Lower panels of A show GLP-1 and BrdU double positive cells whereas upper panels of A show GLP-1 positive BrdU negative cells (highlighted with dotted circles). Scale bar represents 10 μm. Representative immunostaining (n=6-8) of cleaved caspase-3 positive cells in colon **(B)** of mice fed control (CD) or high-fat diet (HFD) for 2 weeks. Scale bar represents 20 μm.

**REFERENCE**

1. Lan A, Bruneau A, Bensaada M *et al.* (2008) Increased induction of apoptosis by Propionibacterium freudenreichii TL133 in colonic mucosal crypts of human microbiota-associated rats treated with 1,2-dimethylhydrazine. *The British journal of nutrition* **100**, 1251-1259.
